# Longitudinal impact of diabetes education program on adolescents’ knowledge and prevention

**DOI:** 10.1097/MD.0000000000047289

**Published:** 2026-01-23

**Authors:** Syed D. Hussain, Shaun Sabico, Mohamed A. Elsaid, Gamal M. Saadawy, Abdullah M. Alnaami, Nasser M. Al-Daghri

**Affiliations:** a Chair for Biomarkers of Chronic Diseases, Department of Biochemistry, College of Science, King Saud University, Riyadh, Saudi Arabia.

**Keywords:** educational intervention, knowledge, prevention, T2DM

## Abstract

This study evaluated the effects of a year-long educational program on adolescents’ knowledge of type 2 diabetes mellitus (T2DM). In this longitudinal study of 643 Arab adolescents (300 boys, 343 girls; mean age 14.8 ± 1.7 years), baseline education sessions were conducted in schools, focusing on T2DM risk factors, development, and the importance of lifestyle modifications. Educational sessions were held every 3 months by certified endocrinologists and nutritionists. Knowledge about T2DM was assessed using the modified Michigan Diabetes Research and Training Center Diabetes knowledge test. A significant increase in T2DM knowledge scores was observed in all participants (40.5 ± 15.2 vs 28.4 ± 15.3, *P* < .001) after 12 months, with significant improvements for both males and females (*P*-values < .001). Girls showed a greater increase in knowledge score than boys (*P* < .001). T2DM participants also improved their knowledge scores significantly (45.4 ± 13.9 vs 28.1 ± 14.6, *P* < .001). After 12 months, 10.3% of participants with normal glycemia transitioned to prediabetes, and 8.3% progressed to T2DM. Among those with prediabetes at baseline, 87.2% achieved normal blood sugar levels, while 55.0% of T2DM participants at baseline reverted to normal. Health education translated to improved knowledge related to T2DM among adolescents, particularly in girls and those with T2DM. Strengthening health education in schools across Saudi Arabia is advisable to raise awareness and prevention of T2DM.

## 1. Introduction

The prevalence of diabetes is on the rise globally, spanning across both developed and developing nations.^[1,2]^ The International Diabetes Federation Diabetes Atlas 10th edition stated that approximately 1 in 6 adults, or 73 million individuals, are currently living with diabetes in the Middle East and North Africa.^[3,4]^ Furthermore, this figure is projected to increase to 95 million by 2030 and 136 million by 2045. Alarmingly 1 in 3 adults with diabetes in this region remains undiagnosed. Diabetes was responsible for 796,000 deaths across the Middle East and North Africa in 2021.^[3,4]^

The Kingdom of Saudi Arabia (KSA) is witnessing a concerning rise in diabetes prevalence among its youth, ranking second in the Middle East and seventh globally according to the World Health Organization.^[3,5–7]^ Unhealthy dietary habits often persist from childhood into adulthood,^[8]^ so promoting high-quality nutrition during early developmental stages is essential for fostering lasting healthy behaviors. Schools play a vital role in this endeavor by serving as an ideal environment for educational initiatives. Their capacity to reach large and diverse groups of young people makes them particularly effective for such programs. By integrating a health education curriculum that emphasizes the importance of proper nutrition and regular physical activity, schools can support students in adopting and maintaining healthier habits concerning their nutrition and physical activity.^[9,10]^ Enhancing knowledge through educational interventions may lead to improved dietary habits and better food selections among participants who receive such education.^[11,12]^

While there has been notable advancement in diabetes prevention strategies aimed at adults, there remains a lack of comprehensive understanding regarding effective approaches for preventing diabetes in adolescents.^[13]^ Previous research has demonstrated that structured training and education positively influences metabolic regulation in individuals with type 2 diabetes mellitus (T2DM). Kocak et al^[14]^ reported improved longitudinal clinical outcomes among T2DM patients in a university clinic, while Ernawati et al^[15]^ showed that diabetes self-management education effectively enhances knowledge, self-care behaviors, and glycemic control in adults with T2DM. Therefore, investigating the impact of educational programs on adolescents is important since early intervention can enhance disease management, encourage healthy habits, and prevent long-term complications.

There are limited studies focusing on the promotion of healthy behavior in school students, particularly in KSA. The aim of this paper is to evaluate the effectiveness of a 12-month educational intervention in improving diabetes knowledge and its role in maintaining glycemic control among Arab adolescents with varying glycemic status at baseline.

## 2. Materials and methods

### 2.1. Participants

**Figure 1.**
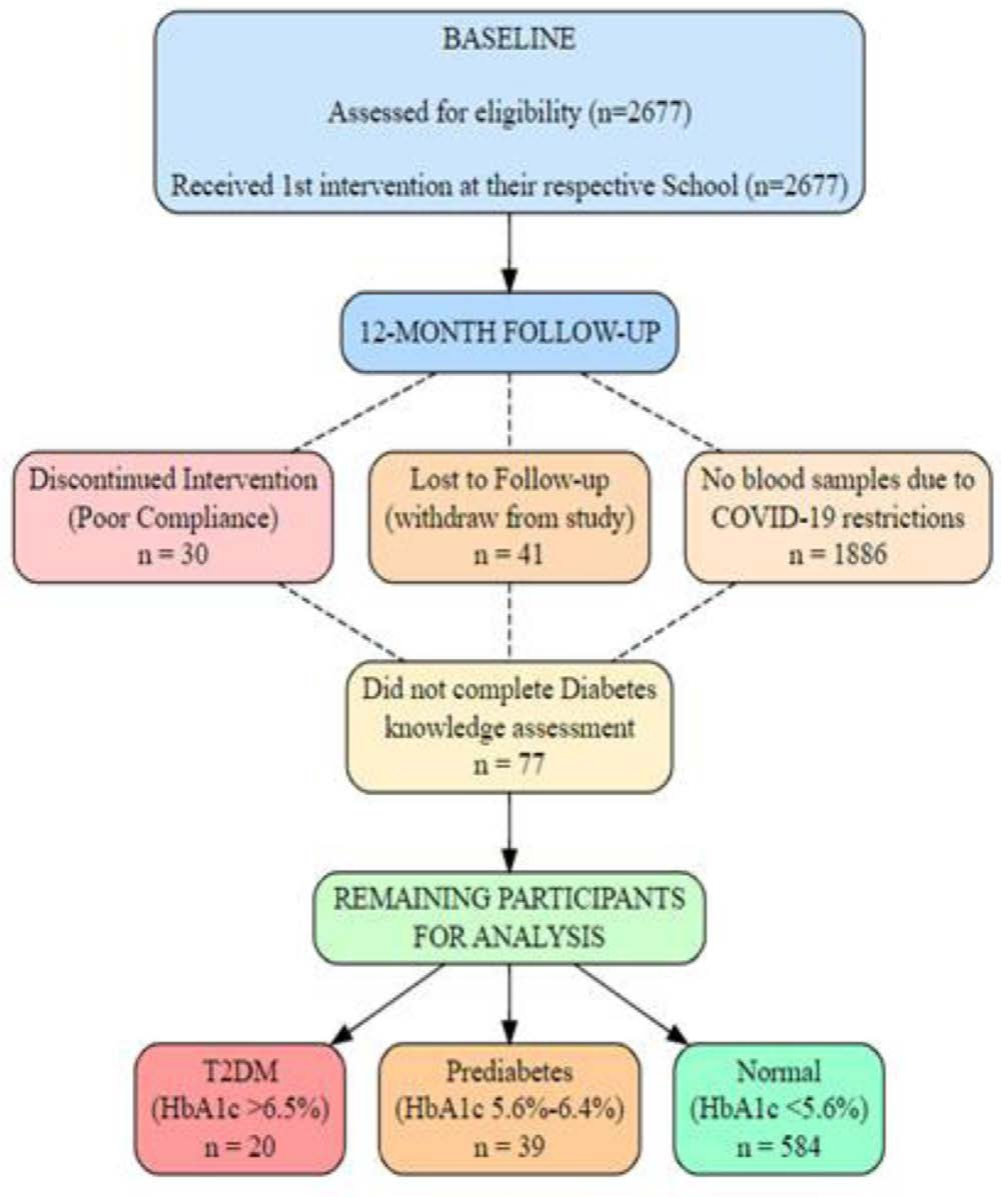
Flowchart.

At the beginning of the study, a cohort of 2677 Saudi children and adolescents, aged between 12 and 18 years (58% girls), were recruited from 60 randomly chosen preparatory and secondary schools located in Riyadh City, KSA.^[16,17]^ This project was conducted from 2019 until 2021 in partnership with the Chair for Biomarkers of Chronic Diseases at King Saud University (KSU) and the Saudi Charitable Association of Diabetes. The study included apparently healthy participants regardless of their weight and glycemic status. Out of a total of 2677 participants, only 704 successfully completed the intervention, resulting in a dropout rate of 73.7%.^[16,17]^ Additionally, 643 participants were able to complete the diabetes knowledge assessment (see Fig. 1).

### 2.2. Biochemical and anthropometric analysis

Anthropometric measurements and fasting blood samples were collected at baseline and 12 months post-intervention by trained coordinators. Measurements included weight, height, waist and hip circumference, and blood pressure. Fasting blood samples were analyzed for lipid profile, glucose, and HbA1c using standardized automated systems including Konelab 20XT (Thermo Scientific, Vantaa, Finland) and D-10 Hemoglobin Testing System (Bio-Rad Laboratories, Hercules).^[16,17]^ Participants were categorized based on HbA1c results using standard diagnostic cutoffs: normal (<5.7%), prediabetes (PD; 5.7%–6.4%), and T2DM (≥6.5%).^[17]^

### 2.3. Diabetes knowledge test

The primary outcome of this study was diabetes knowledge, evaluated at baseline and after a 12-month intervention. Knowledge was measured using a concise, modified version of the Brief Diabetes Knowledge Test, comprising 13 or 22 questions depending on insulin dependence.^[18]^ The original test demonstrated readability at a sixth-grade level based on the Flesch–Kincaid assessment, ensuring suitability for adolescents aged around 12 years.^[18]^ Although not previously used as an outcome measure in educational interventions, this tool shows strong potential for group comparisons and for monitoring knowledge progression over time.

### 2.4. Intervention

All educational intervention sessions were conducted within the campus. Certified dietitians provided participants with standardized guidance on diet and exercise. This instruction covered eliminating juice and soda from their diets, bringing low-fat meals to school and reducing portion sizes. The program discouraged sedentary behaviors while promoting physical activities that the children enjoyed. To ensure educational uniformity across all locations the research team trained all involved staff and provided them with identical instructional materials. Participants were monitored over a 12-month period with follow-up assessments conducted every 3 months.^[16,17]^

Students attended an initial educational session in their classrooms. This session covered type 2 diabetes (T2DM), risk factors, disease mechanisms, as well as the importance of dietary changes and physical activity in delaying its onset. Participants were encouraged to adopt healthier lifestyles by transitioning to a nutritious diet and engaging in regular exercise, as well as losing weight if they fell into the overweight or obese categories. They received information about the recommended lifestyle modifications through various formats, including pamphlets, booklets, infographic videos, and gamification.^[16,17]^

Every 3 months, participants continued to receive education about the lifestyle changes necessary to prevent T2DM. These sessions were facilitated by certified endocrinologists and nutritionists, originally intended to take place at the schools. However, due to COVID-19 lockdowns, follow-up educational activities were conducted via virtual platforms such as Zoom, as well as through social media channels including WhatsApp, Telegram, Facebook, and Twitter.^[16,17]^

For participants with T2DM or PD, personalized lifestyle modifications were provided based on their individual circumstances, communicated through phone consultations and monitored via a diary maintained by a registered dietitian. The suggested lifestyle changes were consistent with those implemented in various diabetes prevention programs across KSA, which included: achieving at least a 5% reduction in body weight, limiting total fat intake to 30% of total energy (with saturated fat comprising no more than 10%), increasing fiber consumption to 15 g per 1000 kcal, and engaging in at least 150 minutes of moderate-intensity exercise per week (or 30 minutes per day).^[16,17]^

Participants were informed about how exercise can aid in regulating blood glucose levels and were prescribed a regimen of aerobic activity lasting 30 minutes, 5 times a week (e.g., biking, swimming, playing badminton, or walking), as appropriate given the restrictions imposed by the COVID-19 pandemic. The frequency, duration, and type of exercise were tailored to fit each participant’s health status and lifestyle.^[16,17]^

### 2.5. Data analysis

The data analysis was conducted using SPSS software (version 22.0, Chicago). Quantitative variables were reported as mean ± standard deviation. Categorical data were summarized using frequencies and percentages. To assess the normality of continuous variables, the Kolmogorov–Smirnov test was employed. Individual responses for each component of the diabetes knowledge test were compared using McNemar test for dependent proportions, stratified by glycemic status (normal, PD, and diabetes). The overall knowledge score was analyzed using a paired *t*-test. Stratification by gender and glycemic status were done to address confounding effect and analysis was done using repeated-measures ANOVA, with post hoc adjustments for multiple comparisons. Statistical significance was set at *P* < .05 (two-tailed).

## 3. Results

Table 1 presents the baseline characteristics of the study participants (N = 643). The mean age was 14.8 ± 1.7 years, with a mean BMI of 21.5 ± 5.7 kg/m². The sample comprised slightly more females (53.3%, n = 343) than males (46.7%, n = 300). Glycemic control, as measured by HbA1c, averaged 5.1 ± 0.6%. The majority of participants (90.8%, n = 584) had normal glucose metabolism, while 6.1% (n = 39) were prediabetic (PD) and 3.1% (n = 20) had T2DM. The Diabetes Knowledge Test results presented in the Table 2 according to 3 distinct groups: individuals with normal health status, those with PD, and those with T2DM. The data is categorized by baseline and follow-up assessments, allowing for a comparative analysis of knowledge acquisition over time. Accordingly, the most significant gains were concentrated on critical questions regarding complication management – specifically sick day protocols (Q15) and signs of ketoacidosis (Q14), and symptoms of nerve disease (Q12). Some topics like the effect of skipping breakfast after insulin (Q20) showed decreased knowledge. These areas highlight complex concepts that require reinforced teaching.

**Table 1:** Baseline characteristics of study participants.

Characteristic	Mean ± SD
N	643
Age (yr)	14.8 ± 1.7
BMI (kg/m²)	21.5 ± 5.7
Sex	
Female	343 (53.3)
Male	300 (46.7)
HbA1c	5.1 ± 0.6
Glucose metabolism status	
Normal	584 (90.8)
Prediabetes (PD)	39 (6.1)
T2DM	20 (3.1)

Data presented as mean ± SD for continuous variables and N (%) for categorical variables.

SD = standard deviation, T2DM = type 2 diabetes mellitus.

**Table 2:** Descriptive statistics of correct responses on diabetes knowledge test according to T2DM status.

Diabetes knowledge test	Non-DM			PD			T2DM		
	Baseline	Follow-up	*P*-value	Baseline	Follow-up	*P*-value	Baseline	Follow-up	*P*-value
1.Which of the following is highest in carbohydrate? Answer: baked potato	127 (21.7)	172 (29.5)	.001	10 (25.6)	12 (30.8)	.815	4 (20.0)	4 (20.0)	1.000
2.Which of the following is highest in fat? Answer: low fat milk	111 (19.0)	159 (27.2)	.001	3 (7.7)	11 (28.2)	.057	3 (15.0)	6 (30.0)	.453
3. Which of the following is a “free food?” Answer: food < 200 cal. Per serving	198 (33.9)	192 (32.9)	.765	9 (23.1)	8 (20.5)	1.000	7 (35.0)	6 (30.0)	1.000
4.Glycosylated hemoglobin (hemoglobin Al) is a test that is a measure of your average blood glucose level for the past: Answer: 6–10 wk	64 (11.0)	87 (14.9)	.058	6 (15.4)	6 (15.4)	1.000	3 (15.0)	4 (20.0)	1.000
5.Which is the best method for testing blood glucose? Answer: blood testing	224 (38.4)	343 (58.7)	<.001	17 (43.6)	25 (64.1)	.134	5 (25.0)	11 (55.0)	.070
6.What effect does unsweetened fruit juice have on blood glucose? Answer: raises it	206 (35.3)	327 (56.0)	<.001	19 (48.7)	20 (51.3)	1.000	7 (35.0)	12 (60.0)	.227
7.Which should not be used to treat low blood glucose? Answer: 1 cup diet soft drink	136 (23.3)	90 (15.4)	.001	11 (28.2)	9 (23.1)	.815	6 (30.0)	7 (35.0)	1.000
8.For a person in good control, what effect does exercise have on blood glucose? Answer: lowers it	105 (18.0)	128 (21.9)	.102	9 (23.1)	13 (33.3)	.481	3 (15.0)	6 (30.0)	.508
9.Infection is likely to cause: Answer: an increase in blood glucose	150 (25.7)	283 (48.5)	<.001	12 (30.8)	19 (48.7)	.167	2 (10.0)	10 (50.0)	.008
10.The best way to take care of your feet is to: Answer: look at and wash them each day.	329 (56.3)	328 (56.2)	1.000	23 (59.0)	24 (61.5)	1.000	13 (65.0)	13 (65.0)	1.000
11.Eating foods lower in fat decreases your risk for: Answer: heart disease	125 (21.4)	291 (49.8)	<.001	8 (20.5)	21 (53.8)	.007	5 (25.0)	13 (65.0)	.021
12.Numbness and tingling may be symptoms of: Answer: nerve disease	215 (36.8)	264 (45.2)	.004	8 (20.5)	18 (46.2)	.031	7 (35.0)	10 (50.0)	.508
13.Which of the following is usually not associated with diabetes: Answer: lung problems	170 (29.1)	390 (66.8)	<.001	7 (17.9)	24 (61.5)	.002	8 (40.0)	16 (80.0)	.077
14.Signs of ketoacidosis include: Answer: vomiting	106 (18.2)	318 (54.5)	<.001	5 (12.8)	15 (38.5)	.021	4 (20.0)	14 (70.0)	.006
15.If you are sick with the flu, which of the following changes should you make? Answer: test for glucose and ketones more often	140 (24.0)	379 (64.9)	<.001	11 (28.2)	22 (56.4)	.019	4 (20.0)	15 (75.0)	.003
16.If you have taken intermediate-acting insulin (NPH or Lente), you are most likely to have an insulin reaction in: Answer: 6–12 h	249 (42.6)	228 (39.0)	.251	18 (46.2)	17 (43.6)	1.000	11 (55.0)	8 (40.0)	.508
17.You realize just before lunch time that you forgot to take your insulin before breakfast. What should you do now? Answer: Check your blood glucose level to decide how much insulin to take	78 (13.4)	217 (37.2)	<.001	2 (5.1)	11 (28.2)	.012	1 (5.0)	6 (30.0)	.063
18.If you are beginning to have an insulin reaction, you should: Answer: drink some juice	70 (12.0)	84 (14.4)	.258	7 (17.9)	8 (20.5)	1.000	4 (20.0)	1 (5.0)	.375
19.Low blood glucose may be caused by: Answer: too much insulin	201 (34.4)	224 (38.4)	.168	15 (38.5)	18 (46.2)	.648	6 (30.0)	10 (50.0)	.388
20.If you take your morning insulin but skip breakfast your blood glucose level will usually: Answer: decrease	103 (17.6)	50 (8.6)	<.001	10 (25.6)	5 (12.8)	.267	3 (15.0)	1 (5.0)	.500
21.High blood glucose may be caused by: Answer: not enough insulin	220 (37.7)	254 (43.5)	.051	15 (38.5)	18 (46.2)	.629	9 (45.0)	10 (50.0)	1.000
22.Which one of the following will most likely cause an insulin reaction: Answer: heavy exercise	177 (30.3)	327 (56.0)	<.001	13 (33.3)	25 (64.1)	.017	7 (35.0)	15 (75.0)	.039

Data presented as N(%) for correct responses; *P*-value obtained from McNemar test; *P*-value < .05 considered significant.

PD = prediabetes, T2DM = type 2 diabetes mellitus.

### 3.1. Overview of knowledge improvement

#### 3.1.1. Normal group

After 12 months, 10.3% (60 out of 584) participants with baseline normal glycemia transitioned to a PD state, while 49 (8.3%) progressed to being T2DM. In the normal group, there is a clear and significant improvement in diabetes knowledge from baseline to follow-up across several questions. For instance, Question 1, which asks about the highest carbohydrate source, shows an increase in correct responses from 127 (21.7%) to 172 (29.5%; *P* = .001). Similarly, Question 5, regarding the best method for testing blood glucose, demonstrates a substantial rise from 224 (38.4%) to 343 (58.7%; *P* < .001), suggesting that educational interventions have effectively enhanced understanding of blood glucose monitoring. The overall trend in the normal group reflects a growing awareness of diabetes-related knowledge, which is crucial for preventing the onset of diabetes and promoting healthier lifestyle choices.

#### 3.1.2. PD group

Among the 39 participants who had PD at baseline, 34 (87.2%) progressed to having normal blood sugar levels, while 3 (7.7%) developed T2DM. The PD group exhibits a mix of results in terms of knowledge gains, with some questions showing significant improvements while others do not. For example, Question 11, which addresses the relationship between low-fat diets and risk reduction, shows a notable increase from 8 (20.5%) at baseline to 21 (53.8%) at follow-up (*P* = .007). However, in Question 2, which inquired about the highest fat source, indicated that while there was an increase in correct responses, it did not reach statistical significance (*P* = .057).

#### 3.1.3. T2DM group

The 11 (55.0%) out of the 20 T2DM participants at baseline experienced a reversal to normal blood sugar levels, and 5 (25.0%) shifted to a PD state. The T2DM group shows significant improvements in diabetes knowledge, particularly in areas critical for managing their condition. For instance, Question 15, which asks about necessary changes when sick with the flu, reveals a dramatic increase from 4 (20.0%) to 15 (75.0%; *P* = .003). Additionally, Question 14, concerning signs of ketoacidosis, shows a significant rise from 4 (20.0%) to 14 (70.0%; *P* = .006), highlighting an increased awareness of serious complications associated with diabetes. These results suggest that educational interventions are particularly effective for individuals with T2DM, equipping them with essential knowledge to manage their condition more effectively.

### 3.2. General and insulin scores according to health status

The general score reflects participants’ overall knowledge and understanding of diabetes-related concepts. Table 3 shows that at baseline, the mean score was 28.4 ± 15.3, which increased significantly to 40.5 ± 15.2 at follow-up (*P* < .001). This substantial improvement suggests that the interventions implemented during the study were effective in enhancing participants’ understanding of diabetes management. The insulin score assesses participants’ knowledge related to insulin management, which is crucial for effective diabetes care. The overall insulin score increased from 25.7 ± 16.3 at baseline to 39.7 ± 16.6 at follow-up (*P* < .001) as reported in Table 3. This improvement indicates that participants gained a better understanding of insulin’s role in diabetes management, which is essential for maintaining optimal blood glucose levels.

**Table 3:** Baseline and follow-up mean correct responses of general and insulin scores according to gender and health status.

Group	General score			Insulin score		
	Mean correct response ± SD			Mean correct response ± SD		
	Baseline	Follow-up	*P*-value	Baseline	Follow-up	*P*-value
All	28.4 ± 15.3	40.5 ± 15.2	<.001	25.7 ± 16.3	39.7 ± 16.6	<.001
Male	23.4 ± 14.9	33.3 ± 12.0	<.001	21.8 ± 17.8	35.4 ± 16.2	<.001
Female	32.7 ± 14.2	46.7 ± 14.9	<.001	29.1 ± 13.9	43.5 ± 16.0	<.001
Normal	28.4 ± 15.4	40.2 ± 15.5	<.001	25.6 ± 16.2	39.6 ± 16.6	<.001
PD	28.0 ± 14.4	41.4 ± 11.2	<.001	27.4 ± 18.0	39.6 ± 17.1	.002
T2DM	28.1 ± 14.6	45.4 ± 13.9	<.001	27.2 ± 14.6	44.4 ± 16.5	.007

Data presented as mean ± SD; *P*-value obtained from independent *t*-test and ANOVA; *P* < .05 considered significant.

PD = prediabetes, SD = standard deviation, T2DM = type 2 diabetes mellitus.

The analysis of the general score by health status as reported in Table 3, reveals that individuals with normal HbA1c levels improved from 28.4 ± 15.4 to 40.2 ± 15.5 (*P* < .001). Similarly, the PD group showed a significant increase from 28.0 ± 14.4 to 41.4 ± 11.2 (*P* < .001), while the diabetes group experienced the most substantial improvement, rising from 28.1 ± 14.6 to 45.4 ± 13.9 (*P* < .001). These findings show the effectiveness of the interventions across varying levels of glycemic control, suggesting that education can significantly benefit all groups, particularly those managing chronic conditions.

The analysis of the insulin score in relation to health status as reported in Table 3, revealed that individuals with normal HbA1c levels improved from 25.6 ± 16.2 to 39.6 ± 16.6 (*P* < .001). The PD group also showed a significant increase from 27.4 ± 18.0 to 39.6 ± 17.1 (*P* = .002). Notably, the diabetes group, while starting with a similar baseline score as the PD group, experienced a rise from 27.2 ± 14.6 to 44.4 ± 16.5 (*P* = .007). This indicates that education on insulin management is particularly crucial for those with established diabetes, as they may face more complex challenges in managing their condition.

Table 4 shows the changes in knowledge scores according to health status. For the general score, individuals with diabetes showed the highest mean change of 17.3 ± 18.0, followed by those with PD at 13.4 ± 19.0, and those with normal HbA1c at 11.8 ± 20.1 (*P* = .434). Similarly, for insulin score, diabetes group had the highest mean change of 17.2 ± 25.6, while the normal HbA1c group had 14.0 ± 22.3 and the PD group had 12.3 ± 22.5 (0.724).

**Table 4:** Mean change in correct responses of general and insulin scores according to gender and health status.

Group	Mean changes in GS score		Mean change in insulin scores	
Male	9.9 ± 19.0	0.011	13.6 ± 24.1	0.684
Female	13.9 ± 20.7		14.3 ± 20.8	
Normal Hba1c	11.8 ± 20.1	0.434	14.0 ± 22.3	0.723
PD	13.4 ± 19.0		12.3 ± 22.5	
T2DM	17.3 ± 18.0		17.2 ± 25.6	

Data presented as mean ± SD; *P*-value obtained from independent *t*-test and ANOVA; *P* < .05 considered significant.

PD = prediabetes, T2DM = type 2 diabetes mellitus.

### 3.3. General and insulin scores according to gender

Both males and females showed significant improvements in their general score as reported in Table 3. Males increased their score from 23.4 ± 14.9 to 33.3 ± 12.0 (*P* < .001), while females showed a more substantial increase from 32.7 ± 14.2 to 46.7 ± 14.9 (*P* < .001). Furthermore, males improved their insulin score from 21.8 ± 17.8 to 35.4 ± 16.2 (*P* < .001), while females increased their score from 29.1 ± 13.9 to 43.5 ± 16.0 (*P* < .001). Similar to the general score, females showed a more significant improvement in their insulin score, reinforcing the notion that targeted educational strategies may be particularly effective for women.

Table 4 shows the changes in knowledge score according to gender. For the general score, males exhibited a mean change of 9.9 ± 19.0, while females demonstrated a greater mean change of 13.9 ± 20.7 (*P* = .011). This suggests that females experienced a more substantial improvement in their understanding of diabetes-related concepts relative to males. The mean changes in insulin score were 13.6 ± 24.1 for males and 14.3 ± 20.8 for females showing that insulin management knowledge is similar between genders (*P* = .684).

## 4. Discussion

The present study demonstrated a significant increase in diabetes knowledge scores in participants after 12 months of participation in the school-based educational program. This increase was more prominent among females and participants with T2DM, suggesting that structured educational exposure is associated with enhanced diabetes-related knowledge among school-aged children. However, given the observational and uncontrolled nature of this study, these findings should be interpreted as associations rather than causal effects.

This study builds on existing research by showing similar trends in the Gulf Cooperation Council context where research on the outcomes of diabetes education for younger groups is scarce. The significant improvements observed in knowledge scores shows the potential value of educational programs in improving awareness and understanding of diabetes, particularly among children with T2DM.^[19]^ These findings align with prior work in Saudi Arabia highlighting the critical need for early preventive education in nation facing increasing number of diabetes patients that are projected to reach 8.4 million by 2030.^[19–21]^

Previous studies in both adult and adolescent populations have demonstrated positive associations between diabetes education and improved knowledge or self-management confidence. Recent systematic review conducted by Riangkam et al^[22]^ concluded that educational intervention enhances knowledge, attitudes, and self-management practices, and improve quality of life. Al-Nozha et al^[23]^ in their cross-sectional study found that education improved knowledge in T2DM and that higher knowledge was associated with fewer diabetes complications. Furthermore, Al-Harbi et al^[24]^ reviewed self-management programs among adults with T2DM and found associations with greater self-efficacy and better glucose control. In the present study the increase in mean total knowledge score from 28.4 to 40.5 aligns with these findings but the lack of a randomized controlled design prevents to attribute the observed improvement entirely to the educational program. Other unmeasured factors such as repeated testing or classroom exposure or broader health campaigns could also have contributed to the observed gains.

The observed improvement among T2DM participants especially in insulin-related knowledge highlights the importance of providing targeted content for individuals living with the condition. In their systematic review, Riangkam et al^[22]^ highlighted that enhanced knowledge of insulin administration supports better understanding of glycemic control in adults. Al-Sabbah et al^[25]^ noted that enhanced knowledge of insulin administration and its role in blood glucose control is also vital for children with diabetes. In our sample children with T2DM showed the greatest improvement which may indicate higher initial motivation and greater relevance of the material. Nonetheless the improvement in blood sugar outcomes among T2DM participants indicates that the increase in knowledge may have contributed to better self-management and clinical results as 11 of the 20 individuals with T2DM achieved normal blood sugar levels and 5 transitioned to a PD state.

Gender-based differences were also observed as females showed significantly higher gains in both general and insulin knowledge scores. This finding parallels earlier research that reported higher health knowledge among female students. Alkhaldi et al^[26]^ also reported that female participants had significantly more correct responses on DM general knowledge than male. Alreshidi et al^[27]^ reported that female students often showed higher engagement levels in School-based Asthma Health Education Program explaining their greater knowledge acquisition. While the current study was not designed to explain these gender differences but potential factors could include higher engagement levels or differential learning preferences. Future research should investigate these psychosocial and educational factors to design more tailored interventions and reduce differences in learning outcomes between genders.

Although this discussion highlights several promising associations, it is important to interpret the conclusions cautiously due to the study’s methodological limitations. First, the absence of a control group or randomization prevents drawing any conclusions about causality. Second, the study relied on a brief knowledge test that may not fully capture conceptual understanding, behavioral competencies or application of knowledge. Third, test–retest effects may have inflated post-intervention scores and factors such as socioeconomic status, school quality or teacher engagement could have confounded the observed improvements. Future research should therefore use more rigorous controlled designs with longer follow-up periods and assess both behavioral and physiological outcomes to determine the practical impact of knowledge improvements. Qualitative methods could also provide deeper insights into learner engagement motivational factors and barriers to applying knowledge. Moreover, developing educational tools that are sensitive to culture and gender may help optimize learning for diverse student groups.

## 5. Conclusions

In conclusion, this study contributes to the growing body of literature emphasizing the importance of diabetes education in the KSA. The significant improvements in diabetes knowledge and management skills among school-aged children highlight the potential of educational interventions to enhance health outcomes. Although educational initiatives appear promising for enhancing diabetes awareness among youth but further controlled research is necessary to determine whether such improvements translate into sustained behavioral and health outcomes.

## Acknowledgments

The authors are thankful to research coordinators who helped in screening of participants, blood samples, and data collection.

## Author contributions

**Conceptualization:** Syed D. Hussain, Nasser M. Al-Daghri.

**Formal analysis:** Syed D. Hussain.

**Funding acquisition:** Nasser M. Al-Daghri.

**Investigation:** Mohamed A. Elsaid, Gamal M. Saadawy, Abdullah M. Alnaami.

**Methodology:** Mohamed A. Elsaid, Gamal M. Saadawy, Abdullah M. Alnaami.

**Supervision:** Shaun Sabico.

**Writing – original draft:** Syed D. Hussain.

**Writing – review & editing:** Shaun Sabico, Mohamed A. Elsaid, Gamal M. Saadawy, Abdullah M. Alnaami, Nasser M. Al-Daghri.
